# The Molecular *vs*. the Dynamic Theory of Life

**Published:** 1897-07

**Authors:** J. Henry Allen

**Affiliations:** Chicago, Ill.


### THE MOLECULAR *versus* THE DYNAMIC THEORY OF LIFE.

J. Henry Allen, M. D., Chicago, Ill.

The philosophy of to-day does not tear down, does not destroy the truth, but builds for us magnificent temples of the truth, where we can securely store its archives. Man's conception of the truth is becoming more positive, more polar, more cosmic, and less anthopomorphic; being heirs to the files of all the ages, we are able to compare, classify, and weigh in the balance of justice the many grand though oft conflicting theories of our forefathers in science, for which we will ever be indebted. But as we saw the materialistic philosophy of the Greeks, which was the kindergarten of the beginning of things leaving us, we had to construct a new philosophy, clothed with dynamics and not a mere organon of scientific methods, but a true synthesis of truth concerning the unknown. How did we construct this true synthesis, by the inductive method of reasoning? We reason downward and backward from the phenomena present to the antecedent phenomena, from effect to cause, from the known to the unknown, from the visible to the invisible, from the finite to the infinite, from the material to the non-material, or the forces, and from the forces to the law that governs the material and the non-material, and last of all from law to the absolute, which was the *a priori*, or the genesis of our inductive method. So in our study of all the subjects coming under the head of philosophy and the sciences, we come to that positive assurance by this inductive method. Hahnemann saw this fact clearly as he studied the great problems of life and disease, in their relationship to physical law, to causation and to cure, and it was by this method alone that he was able to give to the world not alone a true law of biogenesis, but the discovery of a new law, even the law of *Similia*, that has proven to be the only law that is in harmony with this law of biogenesis. So in this paper we will attempt to study life by this inductive method from the two standpoints which head our paper. Under the first heading, which we have designated the Molecular, or the Materialistic, View of Life, which forms the basis of the large school of Homeopathy, which we say is not founded purely upon the teachings of the great founder of homeopathics, nor yet based, but to a very limited degree, upon his teachings as laid down in his *Organon.* The great hindrance to the progress of this school is in their not accepting Hahnemann's theories, and from this fact alone have they become purely materialistic, only seeing matter or the material as acting on matter, or the material. Yet we are told in Paul's Epistle to the Romans that the things that we see are not made of the things that do appear. Are not all things in the first place thinkings, and were not all material things evolved out of the forces? Is not law force and motion, not only the original genesis of matter, but the maintainers of matter? It is not my intention to condemn or to try to set aside the molecular theory of life, but simply to show some of its inconsistencies. In the first place, molecules cannot act of their own accord, they are powerless to change their own condition, a fact we know to be true according to the first law of motion concerning the inertia of matter. What do we imply by molecule; is it not a synonym for an exceedingly small mass of matter as much subject to the law of motion as if it were a mountain unable to move or change its position unless acted upon by force? Strange beings these elemental molecules, and of the sixty or seventy that we are acquainted with no two are alike, each has its own peculiar angles and facets, its color, its characteristics, and when we come to them science closes its gates and we are brought face to face with the infinite. We know not how they came, but we do know that all nature in the material universe is made from them, and that all matter moves by virtue of the same forces that move them, and that it is the business of the forces and the laws that govern them to handle matter in all its forms, whether it be in the organized or unorganized state. Now, what is true of matter in the organized state is true of matter in the unorganized state, as it is true that the law of crystallization is the architect as well as the builder of the crystal, shaping its angles and its facets, always the same by virtue of the material used in its construction. So it is with biological law, it has the power not only to construct an organism from matter, but to change matter to the nature of itself, and more than that, it must constantly keep in repair that degenerative process which seems so necessary on the negative side of life, and while the biological unit can be chemically analyzed, we must not lose sight of this fact, that it is not the living cell we can bring under the domain of chemistry or the microscope, as it is but the *debris* of life, the husks of life:

> The tiny cell is forlorn,
> Void of its little living will,
> That made it stir on the shore,
> Did he stand at the diamond door
> Of his house in a rainbow frill.

Yes, standing within the diamond door of this house of the soul is something that alchemy has not known, that chemistry is subservient to, whose king is the DYNAMIDES. The physicist and the materialist prove by mathematical computation the size of the material atoms, beyond which there can be no further division, and the chemist joins him in resigning the indivisability of matter to its burial in the sepulchre of the dynamics. The time has passed I think for the explaining of life by hydrostatics or chemistry. Even if such a great scientist as Mr. Dolbear should presume to say that the time would come when the chemist would be so acquainted with protoplasm that he could as it were chemically mix life. Oh! but a little knowledge makes us bold.

Tyndall says we have every reason to believe that nerve force, or nerve transferred to the brain is motion. Joslin said twenty-five years ago that Homeopathy was destined to effect a glorious evolution in the art of healing, and to lead to new views of the constitution of matter, and so his prophecy has been fulfilled. We begin to see the true relationship that exists between our high potencies and the disturbed organism when we prescribe the one hundred thousand potency, as we do frequently to correct some disturbance in the organism; then we know that life is not a chemical change, or rather a molecular change, depending upon chemistry, a theory so comforting to the materialist that life is a material thing whose primary unit is a compound and complex material known by the name of protoplasm, whose formula never has or ever can be written, and that all changes in the man are due to chemical action. But does not our reason tell us that there is something more potent in life than chemistry? The physicist and the materialist can find nothing beyond the twelfth centesimal potency, yet in spite of all that we know there is something left, although we cannot see it with our physical eyes, and that something, by virtue of its sick-making powers, demonstrates to us without doubt that a something more potent than mechanics or chemistry is with us even in our power and even as forces dynamic in their nature are developed from mechanical and from chemical affinity or matter proportionately mixed, so by the power of potentization do we enter the realm of dynamics by, and through this method alone are we able to draw out the spirit-like essence from matter—even the dynamides of God, who must have at creation crowned dynamics the king of law and of motion, and *Similia* the premier of the harmonic forces of the universe. All forces governing matter harmoniously are harmonic forces or similar forces, and all forces that bring disharmony to matter or to motion are dissimilar forces, whether in organized or unorganized matter, and the disturbance, in proportion as they are dissimilar, and the primary force, whether it be life force or physical, has power over it in proportion as it is similar. Thus we reason, returning to our subject; that which is left of the crude drug when potentized to the thirtieth centesimal potency is not a molecule, it is not even an atom, for it has died to matter, and in its death it has been separated from matter, and by virtue of its death to matter it has been born into the force world and now become a peacemaker through similies law, between the life forces and the subversive forces that the life forces have become subservient to. Again, we cannot say that life is due to radiant energy, or to the gravitational forces of our universe, although they have much to do with the evolution or metamorphosis of life, for we have potencies of radiant matter carried forward to that point when even radiant matter is lost sight of, yet their action is as prompt and as positive as if they had been developed from Plumbum or Aluminum. Therefore, we say—and I know my words can be substantiated by a host of living witnesses who have the love of our science at heart, also confirmed by the lives and writing of our sacred dead—that our remedies (potencies) act not by virtue of any molecular power, first because we have demonstrated that they are not molecules, when carried up to the twelfth centesimal potency, therefore cannot act upon the disturbed life force by any molecular process. Secondly, they cannot act through any chemical law, because the thirtieth centesimal potency places the remedy beyond chemistry or chemical law, all action of which is governed by atomic weight and molecular proportion. So you see we have by potentization taken it out of the field of chemistry and destroyed the molecule. Its very character is destroyed, and it has become a force peculiar to itself, for we know that in order to produce a force we must entirely destroy matter, and the greater the destruction the more potent the force; every feature of it must be annihilated before we liberate from it force; its height, its length and breadth, its thickness must be destroyed, and as matter dies to the influence of some other form of matter or force, is it born into the force world, and the liberated force finds its affinity in the new medium that liberated it, but subject to transference through the same medium to the life forces in the body, and if its potens is a similia to any disturbance in that life force it becomes a mediator to any subversion in biological law. When we deal with chemistry we are dealing with molecules, but when we destroy the molecule which is admitted to be the birthplace of the creation of things, by the combination of atoms, the primary units of all, can we any more conceive of it being matter, is it not now a dynamic, and are we not brought face to face with that great truth of our forefathers in science that matter and force are interchangeable, that before it was matter it belonged to the force world, and when it was destroyed it was again force? Does not the death of organized life, let alone matter, tell us the same story, and did not that same force called life take hold of matter with its invisible hands, and construct itself a tabernacle for its temporary physical existence, and as long as they harmonized did demonstrate even all the true manifestations of life, that we see in the body, mind, and spirit, each being subservient to the other in that trifold relationship? Does all this depend on molecular change and molecular power alone; does not common sense and all the powers of reason within us say No; and while we recognize the molecular changes that take place in organized matter, both as to construction and to its disintegration, but knowing they have no power to change themselves we are forced to recognize the prime mover, which we call life, which simply uses the material to fulfill the purpose in this physical world for which the Creator of the soul or life had designed. For the purpose can only be fulfilled in and through the design, the design not being the original thought, but the purpose. Do we not, being materialists, look upon the wrong side of our created bodies for the thing called life? It is not that life was a creation through the body, but that the body was a creation through the life. The thing that we see not being made of the things that do appear, but the thing that does not appear (life), took hold of that which does appear (material) and changed it into that which could fulfill the purpose for which it, the life, was designed, clothing it simply with the material or physical. Now, seeing that this force, or potens, called life, could clothe itself with the physical and construct its physical medium so that it could think, feel, hear, see through it, has it not all power over the physical to control and govern it? Now, if this be true, shall we not centre these forces (remedies) that we know do reharmonize the changed or disturbed organism upon the force or forces that control the organism, and not upon the organism itself, for if we centre them upon the organism is there not danger of disturbing the organism more, and in place of bringing peace we bring war? And again, if we do select the similar force (remedy) and direct it against the disturbed life force, and it be not a minimum force, then do we not destroy the equilibrium in place of establishing it? The biologists of to-day tell us that chemical organization ceases when the protoplasm was formed, yet they also say that it is the basic principle of life, or the highest expressions of life in the chemical series. If this be true, then we are to understand that when life becomes biotic it is not longer a chemical process, but a molecular process. But have we not already stated a chemical process to be a molecular process, and do not our physiologists teach us that all nerve stimuli are chemical action? Motor, sensory, or secretory, is a transference of energy, due to chemical formation and chemical change, caused by vibratory motion of heat and chemical decomposition, modes of motion, etc. Stuff and nonsense. Is not life behind chemical action, and are not these things qualities of life, or the life forces dealing with the physical, a quality inherent in the germinal element or in the *a priori* of life, and in place of the protoplasm bridging the chasm between the organic and the inorganic world, it is life that bridges over the chasm, for we know that biological law has the power to take hold of the organic world and change it into an organism suitable to fulfill and carry out the purpose for which that life (entity) was designed. It also has the power to store up cosmical energy for the future use of that organism. Let us again appeal to induction. Is the body the entity or the soul? which is the life? The entity is the soul or the spirit of the man, and what is true of man is true of all animal or vegetable creation. Yes. Well, then, if the soul is the entity, all power must be given and does belong to the soul, which is the entity, thus the soul having all power, it surely has the power to reproduce itself under the proper conditions, or, in other words, to create new life. Does the body or the soul of the seed or the nut or the comb, produce the new life or the new creation? The soul is the germinal part, of course, the body of the seed dies under the germinal process, and returns the material from which it was constructed to the original owners; what did it do under the germinal process and period of development and growth? It was simply building itself a new physical dwelling-place for a larger and a greater life, and what is true of the vegetable is true of the animal, as we have seen. What did it construct it out of, the physical; you agree to that I know. Then, if that be true it must necessarily be a chemical change, and if a chemical change a molecular one, because all chemical change is due to molecular. So we conclude that chemical change is molecular; both, instead of producing life, is the handiwork of life, or the life forces working with the physical, and through the physical or material, or, in other words, it is the resultant of the life forces, and I have reason to believe that this spirit force or life is a spiritlike organism that is as real as the physical one in which it is tenanted, and that any disturbance of the physical is first due to a disturbance of this dynamic existence. Here are pivotal points, and from whatever stand we take are we either homeopathic or antipathic in our treatment of the sick. If we conceive disease to be a chemical change in the physical or a molecular disturbance, we must treat it antipathically, but if we conceive disease to be a disturbance of the dynamic man, then will we enter the kingdom of the dynamics for the means by which to restore disharmony. For do we not see our physiomedic and physiochemic friends gradually drifting into organismic medicine, not knowing that disease is a disturbance of the life force in the dynamic man, but thinking the pathological change to be due to chemical change in the organism, confining its disturbance to a part, as if the functions of a part could be maintained independent of the whole, when we know it is but a biological unit of that whole. Again, is man a test-tube that we might chemically compound within the organism such ingredients as would make more life or maintain what life there was in that organism. Neither can the organismic doctor supply the organism with the proper chemicals, for the liver is not a retort, and the power of assimilation belongs alone to the life force, and in its restoration it may require something very foreign to any chemical constituent that the liver may lack. And while it seems plausible to the pharmaceutic physician to counteract an acidity of the stomach by the use of *Soda*, yet we know it gives us but palliation, knowing we have but temporarily neutralized the acidity, for the deranged life force is continually making more acid, through the law of overaction, requiring more Soda and stronger doses, which, in the end, not only fails to accomplish their purpose, but fails to longer even palliate. Thus we see that through the chemical phenomena we can only get palliation if we get any results at all. Why? Because we are only dealing with the results of the life forces in the physical instead of the life forces themselves. But what do we find in looking beyond the chemical or molecular man? We find the dynamic man, that invisible entity who when in a normal condition is in harmony with all law, all the forces, all things in the universe of God, but when out of harmony subject to them all, and in proportion as it is disharmonized. This brings us into the presence of a new relationship, of new thought, and the study of life becomes the study of the laws of motion, and the laws of action and reaction. Of the dynamic forces and the laws governing dynamics—for we now conceive of life as being a force—so our whole study of medicine becomes a study of the dynamics of life, of law, and of the forces. Why do I carry this thought so far and dip so deeply into it? Because if I say life is a mechanical process you will want to treat man mechanically when he is sick, and if I say it is a chemical process you must necessarily become a chemical doctor, or if I say life is a molecular process you cannot go beyond the molecule, therefore you cannot leave the material, and if I say that the mind controls all matter, and in place of it being an attribute of life, it is all there is to life, then you will treat it psycologically, or by mind cure. Again, if I say human life is an entity in the hands of its Creator, and is held, sustained, and controlled by a divine fiat, and not governed by and sustained by virtue of its abeyance to physical law, then you will want to treat him by faith cure, or divine healing. But if I say life to be a vital principle governed by true biological law, which, if we live in correspondence with, we are well, and if out of correspondence with, we are sick, and if we fail to correspond at all we die. Therefore our study of life in health will be purely biological dynamics, and our study of it in sickness, biological dynamics, and the study of its function, physiological dynamics, and of the mind psycological dynamics, and the study of its symptoms (disease) as pathological dynamics, and the study of the forces (remedies) by which we restore harmony in the disturbed life forces (therapeutics) pathopoëtic dynamics; so that life, while it is to be studied from the standpoint of all the sciences mentioned, it is to be studied from the standpoint of dynamics only.

Now in conclusion. You see that one of the great differences between the Hahnemannian school of Homeopathy and all other schools of medicine is in the difference of their conception of life. We start from entirely different premises. The dominant schools, starting from matter, inert, life as a chemical process working in and through matter, building up their speculations from anti and post-mortem examinations, from physical and chemical data, with no knowledge of a vital force or its modifications upon the organism, life force, they expel from their physiologies and from their schools, never thinking that without these life forces pathology cannot be called the reverse of physiology, for if physiology is the study of normal function pathology is the study of abnormal function; and as physiology is true function pathology is bad or morbid function, both the results of the workings of the life forces, the former is harmony, the latter out of harmony. The former fulfilling the purpose for which the organism was created, the latter hindering or destroying that purpose. Paragraph 9 of *The Organon* clears all doubts from our minds on this subject. The life force when in harmony governs the organism, develops, repairs, maintains, which is health; a disturbance of which is disease, which is fully revealed by symptoms, and if the pathogenesis is dynamic then the pathopoësis must be dynamic. All these mysteries might yet have remained mysteries had not Hahnemann revealed them to us, and we still be searching among the clods of materialism. But if we would only come to the knowledge of the light we have had revealed to us through Hahnemann, these things would no longer remain a mystery to us, for the revelation is made known through the study of the law. The great inhibitory forces that governed the motions of the whole sidereal universe had remained a mystery had not Newton worked it out in the philosophy of the falling apple, and when gravitation was made known the mystery of the complex motions of the spheres was made known. Old King Solomon could have explained the way of the eagle through the air or the way of the serpent upon the rock had he known gravitation; so we might say of medicine to-day, how can men believe in Homeopathy who know not the law, and how can they believe in potency who have not studied minimum force in its action upon matter. No wonder they cannot harmonize their molecular theory, which belongs to chemistry and not to that dynamic law of life which not only governs all life, but the action of the highest potency ever made by mortal man.

